# Dr. Davis’ “History of Medical Education and Institutions,” and the Reviewer in the N. Y. Journal of Medicine

**Published:** 1851-03

**Authors:** 


### ARTICLE II.

*Dr. Davis's "History of Medical Education and Institutions," and the Reviewer in the New York Journal of Medicine.*

Out of sheer justice to Prof. Davis we give the following from some unknown author who has had acumen enough to see, as every candid observer must, that the critique referred to was instigated by personal feelings against him. The endorsement of the Editor of the New York Register of Medicine and Pharmacy, of the position of his correspondent, contrasts strongly with the course of a certain medical editor out West of us, whose morbid desire to criticise the work, had led him to claim to have received a copy from the author, who informs us he never sent it to him, and with a preface of balderdash quotes the New York Journal's strictures, either not knowing or not caring to know that they were unfair in their representation of the nature and design of the work, and unjust and illiberal in their comments upon it. But as we understand the reviewer is to be reviewed, we shall see whether our cotemporary will have the fairness to give its readers both sides of the question.

*To the Editor of the Register:*

In the January number of *The New York Journal of Medicine*, I notice what purports to be a review of Professor Davis's Work on Medical Education, but which is more strictly an ill tempered tirade upon Dr. Davis's character, charging him with ignorance of the subject he treats, and his work a "*caricature* and a *libel* upon the profession," which the reviewer asserts to be "respectable."

Desirous of knowing how far these charges are correct, I have obtained a copy of the book, and from it I find abundant evidence that critics are liable to magnify trifling errors.

In the first place the writer, in order to show cause for saying what evidently is his first desire, finds it necessary to misrepresent the object of the work by stating it to be a "History of Medicine," whereas the very title of the book restricts it to a "History of Medical Education," and on such ground, charges Dr. Davis with having failed from ignorance in what he not only does not claim, but never intended to do; and which he distinctly states in alluding to medical history proper, that such topics are incidentally introduced, constituting no part of the main object of the work.

In alluding to the second chapter, the reviewer as usual, prefacing what he has to say of the work with reflections upon the author, introduces the following quotation from its last page: "I have not been able to find a single volume on any branch of medical science or practice published by an American physician during the first twenty years after the close of the revolutionary war." And he adds, "This is a precious confession for the historian of our profession, and we give him full credit for his honesty! What will the reader think, however, of Mr. Davis's knowledge, or his competency for the task of an historian, when he is told that the period mentioned by him abounds in original publications;" and then follows a list of sixteen works triumphantly brought in to show the reviewer's learning and the author's ignorance. Now it is hardly possible that there are any who would be deceived by this parade, and wilful misinterpretation of this sentence, notwithstanding it is carefully expressed. It must be remembered that Dr. Davis's book is a *History of Medical Education*, and the works referred to in the above quotation, were only such as are embraced under that head, viz.—works on Physiology, Anatomy, Surgery, Practice, etc. etc., or in other words, "text books," of which there is not one in the list of sixteen introduced by the reviewer." That it was possible for the author of this review to have mistaken the import of the sentence we cannot believe, without we are assured that he does not comprehend the meaning of English words, or else, that he does not tell the truth when he states that he has read the book through, for in the very chapter which he passes over to find this isolated sentence, there is frequent allusions to original works upon general and special subjects, and particularly the "*Medical Inquiries and Observations of Dr. Rush*," with the titles in full and quotations credited to the same; yet this last heads the "list of sixteen" which are brought forward as works which Dr. Davis could never have seen.

There are other points equally at variance with the true spirit of justice and candor; but we trust the examples already given are sufficient to show that the article, in the Journal of Medicine, was instituted by any other than a desire to give an impartial criticism of the merits of the work.

*New York, February*, 1851. ***

[We publish the above communication, although not in order, as the Journal of Medicine is the proper medium for answers to articles it contains; yet as we believe our correspondent has the right of the matter, we do not consider it a good reason why we should refuse his article.—*Ed. Register.*] —*New York Register of Med. and Phar.*

